# Transient receptor potential channel A1 and noxious cold responses in rat cutaneous nociceptors

**DOI:** 10.1016/j.neuroscience.2009.11.065

**Published:** 2010-02-17

**Authors:** J.P. Dunham, J.L. Leith, B.M. Lumb, L.F. Donaldson

**Affiliations:** Department of Physiology and Pharmacology, University of Bristol, School of Medical Sciences, University Walk, Bristol, BS8 1TD, UK

**Keywords:** TRPA1, nociceptor, cold, pain, cinnamaldehyde, capsaicin, CED, cambridge electronic design, CFA, complete Freund's adjuvant, c.v., conduction velocities, DMSO, dimethylsulphoxide, DRG, dorsal root ganglia/ganglion, RF, receptive field, TRP, transient receptor potential, TRPA1, transient receptor potential channel A1, TRPV, vanilloid family of transient receptor potential channels

## Abstract

The role of transient receptor potential channel A1 (TRPA1) in noxious cold sensation remains unclear. Some data support the hypothesis that TRPA1 is a transducer of noxious cold whilst other data contest it. In this study we investigated the role of TRPA1 in cold detection in cutaneous nociceptors *in vivo* using complementary experimental approaches. We used noxious withdrawal reflex electromyography, and single fibre recordings *in vivo*, to test the hypothesis that TRPA1-expressing primary afferents mediate noxious cold responses in anaesthetised rats. TRPV1 and TRPM8 agonists sensitise their cognate receptors to heat and cold stimuli respectively. Herein we show that the TRPA1 agonist cinnamaldehyde applied to the skin in anaesthetised rats did not sensitise noxious cold evoked hind limb withdrawal. In contrast, cinnamaldehyde did sensitise the C fibre-mediated noxious heat withdrawal, indicated by a significant drop in the withdrawal temperature. TRPA1 agonist thus sensitised the noxious reflex withdrawal to heat, but not cold. Thermal stimuli also sensitise transient receptor potential (TRP) channels to agonist. Activity evoked by capsaicin in teased primary afferent fibres showed a significant positive correlation with receptive field temperature, in both normal and Freund's complete adjuvant-induced cutaneous inflammation. Altering the temperature of the receptive field did not modulate TRPA1 agonist evoked-activity in cutaneous primary afferents, in either normal or inflamed skin. In addition, block of the TRPA1 channel with Ruthenium Red did not inhibit cold evoked activity in either cinnamaldehyde sensitive or insensitive cold responsive nociceptors. In cinnamaldehyde-sensitive–cold-sensitive afferents, although TRPA1 agonist-evoked activity was totally abolished by Ruthenium Red, cold evoked activity was unaffected by channel blockade. We conclude that these results do not support the hypothesis that TRPA1-expressing cutaneous afferents play an important role in noxious cold responses.

Thermosensation is important to the well-being of an organism, allowing escape from potentially damaging environments or stimuli, but also contributing to the maintenance of body temperature. The transient receptor potential (TRP) channel family is an extensive group of channels, some of which are gated by temperature and many of which are expressed in primary sensory neurones (Dhaka et al., 2006). Many thermoTRPs are implicated in heat responses (TRPV1, TRPV3 and TRPV4), and two have been identified with cooling or cold responses (TRPM8 and TRPA1).

TRPA1 has been proposed to be a noxious cold sensing cation channel (Story et al., 2003). The evidence for and against TRPA1 as a noxious cold sensor has been extensively reviewed recently (Reid, 2005; Foulkes and Wood, 2007; Caspani and Heppenstall, 2009; Karashima et al., 2009). There is still no consensus on whether TRPA1 is a noxious cold transducer molecule or not. TRPA1 was not initially identified by any thermal gating (Jaquemar et al., 1999), but was only later proposed to function as a noxious cold sensor activated by temperatures circa 17 °C *in vitro* (Story et al., 2003).

*In vitro* experiments on the cold activation of TRPA1 have been complicated by the variability in the degree and rate of cooling applied to the cells, the cell type in which TRPA1 is studied, and the species in which the channel has been studied (reviewed in; Caspani and Heppenstall, 2009; Chen and Kym, 2009; Kwan and Corey, 2009). This has led to reports showing TRPA1 sensitivity to cold in heterologous systems (Story et al., 2003; Bandell et al., 2004), and others that do not (Jordt et al., 2004; Nagata et al., 2005). It was proposed that cold sensitivity in TRPA1 was secondary to another cold sensing mechanism which caused an increase in intracellular calcium which then activated the channel (Doerner et al., 2007; Zurborg et al., 2007), but TRPA1 appears to maintain cold sensitivity in isolated patches and in the absence of calcium (Sawada et al., 2007; Karashima et al., 2009). In sensory neurones (dissociated from dorsal root or trigeminal ganglia), relatively few neurones are cold sensitive (10–25%); (Reid et al., 2002; Viana et al., 2002; Thut et al., 2003; Munns et al., 2007; Karashima et al., 2009), and TRPA1 is difficult to activate by cold, having a slow activation rate (Reid, 2005). Within the population of sensory neurones that respond to temperatures<15 °C, cold sensitivity is not obviously correlated with TRPA1-expression, as assessed by cellular response to TRPA1 agonists, such as mustard oil or cinnamaldehyde (Reid, 2005; Munns et al., 2007), although others report a strong relationship between cold and agonist responses (Sawada et al., 2007; Karashima et al., 2009). These discrepancies may to be due, in part, to weaker calcium responses to cold, compared to mustard oil stimulation in sensory neurones (Karashima et al., 2009). There are, however, clear differences in the cold/TRPA1 responses of spinal and visceral sensory neurones, with a larger proportion of visceral afferent cell bodies showing cold sensitivity (∼50%), of which >80% were responsive to the TRPA1 agonist cinnamaldehyde (Fajardo et al., 2008).

It has been proposed that TRPA1 is “actively suppressed” (Reid, 2005) under normal conditions, and that channel activity may be relieved of suppression under pathological conditions. Again, data in this area are not consistent. Although some reports suggested that cold hypersensitivity in chronic inflammatory or neuropathic pain might be associated with an increase in TRPA1 expression (Obata et al., 2005; Ji et al., 2008), this has been recently contested (Caspani et al., 2007). Inhibition of TRPA1 under pathological conditions, either using intrathecal antisense oligonucleotides (Katsura et al., 2006) or a locally administered antagonist (Petrus et al., 2007) ameliorates neuropathic or inflammatory cold hypersensitivity. These findings suggest that TRPA1 function is modulated under pathological conditions.

The lack of consistent findings using *in vitro*, expression and behavioural approaches has not been greatly clarified by the study of behaviour in TRPA1-deficient mice. In two independent knockout lines, cold sensitivity was either unaffected (Bautista et al., 2006) or slightly reduced but only in female mice (Kwan et al., 2006). Interpretation of these findings may be complicated by the observation of TRPA1 expression in spinal motoneurones and skin (Anand et al., 2008; Atoyan et al., 2009). In addition, the behavioural tests used may contribute to the differences seen; time of cold stimulus applied to paw or tail, and stimulus intensity may all play a part (reviewed in; Kwan and Corey, 2009). For example, TRPA1 knockout animals had very clearly blunted responses in tests with more prolonged exposure to a cold stimulus, or to a shorter exposure to a more extreme stimulus (Karashima et al., 2009).

Despite the plethora of behavioural *in vivo*, and *in vitro* data on TRPA1 and cold, there are very few studies on the contribution of TRPA1 to cold sensing at the neuronal level, *in vivo* (Ji et al., 2007, 2008; Dunham et al., 2008; Pecze et al., 2009). Cultured dorsal root ganglion (DRG) or trigeminal neurones are used as models of intact sensory neurones on the assumption that the molecular receptors normally found at peripheral or central terminals are found on the soma, and confer similar properties to that site, as found in the physiological receptor terminal. This approach has yielded valuable data, and enables, for example, identification of putative nociceptors (Gold et al., 1996), but has disadvantages with respect to investigation into cold sensation. Cultured DRG neurones are axotomised, and may better represent a pathological state, culture conditions may influence channel expression, for example TRPA1 (Anand et al., 2008), and responses to thermal stimulation can be dramatically affected by, for example, peripheral vascular responses or thermal conductivity of surrounding tissues. This is demonstrated by the observation that human cold pain perception is affected by environmental temperature (Strigo et al., 2000). It is therefore vital that observations made *in vitro* are corroborated *in vivo*.

In this study we have investigated the link between cold transduction/sensation of TRPA1 *in vivo*, testing two hypotheses: (1) That if TRPA1 is a cold sensor/transducer *in vivo*, then agonist sensitisation of TRPA1 will sensitise TRPA1 to cold stimulation and *vice versa*, in a manner analogous to sensitisation of the TRPV1 channel (Zimmermann et al., 2005). (2) That cold sensitive afferents will express TRPA1 and that both agonist and cold-evoked responses can be blocked by a TRP channel blocker.

## Experimental procedures

### Animals

All experiments were carried out in accordance with the UK Animals (Scientific Procedures) Act, 1986, and associated guidelines and with approval of the University of Bristol Ethical Review Panel. All efforts were made to minimize both animal numbers and suffering in the experiments. A total of 25 male Wistar rats (250–350 g) were used in these experiments. Animals were given access to food and water *ad libitum* and housed in accordance with UK Home Office regulations. All chemicals and drugs were obtained from Sigma Aldrich, Gillingham, UK unless otherwise specified.

### TRPA1 agonist effects on thermal withdrawal thresholds

Anaesthesia was induced using 4% halothane in O_2_ and maintained using a constant i.v. infusion of alphaxalone/alphadolone through a jugular cannula (Saffan, Schering Plough Animal Health, Welwyn Garden City, UK; 14–27 mg kg^−^ h^−1^). An i.m. bipolar electrode, custom-made from two short lengths of Teflon-coated 0.075 mm diameter stainless steel wire (Advent Research Materials, UK), was inserted into the biceps femoris of the left hind leg to record electromyographic (EMG) activity during the withdrawal reflex. The EMG signal was amplified and filtered, before being captured for subsequent analysis via a CED1401plus (Cambridge Electronic Design, Cambridge UK) onto a PC running Spike2 v5.13 software (CED, UK). Arterial blood pressure and skin/instrument interface temperature of the hindpaw to be stimulated were also captured and stored on the PC. Following initial surgery, anaesthesia was reduced to a level at which animals were moderately responsive to firm pinch of the contralateral forepaw and brushing of the cornea using a cotton swab. Animals were then allowed to stabilise at that level for a minimum of 30 min.

Noxious heat stimulation of the hindpaw, which evoked a withdrawal reflex, was delivered to the dorsal surface using a custom-made lamp system, as described previously (McMullan et al., 2004; Leith et al., 2007). In brief, heat from a sputter-coated projector bulb was focused onto a blackened copper disk positioned at the focal point. A T-type thermocouple (made in-house: 0.02 mm diameter Copper/Constantan, Goodfellow Metals, Huntingdon, UK) was fixed to the outer surface of the copper plate and therefore measured the surface temperature of the skin when placed in firm, even contact with the hindpaw dorsum. Using a constant bulb voltage, a slow rate of heating (2.5±1 °C s^−1^) was used to preferentially activate C-fibre (unmyelinated, capsaicin-sensitive) heat-sensitive nociceptors (McMullan et al., 2004). The cut-off temperature (55 °C) of the heat lamp was controlled via a Spike2 script to prevent tissue damage.

Noxious cold stimulation was delivered using a custom-made Peltier device (surface area ∼2 cm^2^), again placed in firm, even contact with the hindpaw dorsum. The interface temperature of the hindpaw dorsum and Peltier device was recorded using a T-type thermocouple positioned securely between the cooling plate of the device and the hindpaw skin surface. The Peltier:skin interface temperature initially decreased rapidly, reducing from a Peltier holding temperature of ∼30 °C to 0–5 °C in the first 4 s, and thereafter slowed, approaching a plateau at −15 °C. The initial rate of temperature change was approximately 6 °C s^−1^ in the first 4 s, and ∼1 °C s^*−*1^ overall. The cut-off temperature was controlled manually and set to −15 °C.

Heat or cold stimulation was applied at 10 min intervals and the threshold temperature at which the withdrawal reflex occurred was recorded. Once a reproducible baseline of paw withdrawal thresholds had been achieved (up to five ramps, of which the last three were used to calculate baseline withdrawal temperature), dimethylsulphoxide vehicle (100% DMSO, 50 μl) was applied to the dorsal hindpaw surface, stimulation resumed 10 min post-application and paw withdrawal thresholds recorded. Cinnamaldehyde (10% in DMSO, 50 μl) was then applied to the hindpaw surface; again stimulation resumed 10 min post-application and paw withdrawal thresholds were measured over three further trials.

### Teased fibre electrophysiology

Teased fibre recordings from sensory primary afferent fibres (units) were made as previously described (Dunham et al., 2008). Briefly, animals were anaesthetised (60 mg/kg i.p.) and maintained areflexive on sodium pentobarbital (20 mg/kg/h i.v.). The trachea and jugular vein were cannulated to maintain the airway and for anaesthetic delivery respectively. Body temperature was maintained with a feedback controlled heater and rectal thermistor. At the end of all experiments, rats were killed by an overdose of sodium pentobarbital.

The right saphenous nerve was exposed mid-thigh and was isolated from the surrounding tissue. A pool of warmed paraffin oil was made of the surrounding skin to prevent dehydration and, following removal of the epineurium, fine filaments of the saphenous nerve were teased to enable differential recording of neuronal activity via bipolar platinum wire electrodes. Action potentials were amplified and passed through a CED1401 analogue to digital converter. Spikes were recorded and analysed using CED Spike 2 v5 software.

### Close intra–arterial drug administration

Close arterial drug administration (i.a.), was performed as previously described (Dunham et al., 2008). Briefly, drug was delivered to the cutaneous receptive fields (RFs) of units under study via a cannula placed at the bifurcation of the abdominal aorta accessed via the femoral artery of the opposite hind limb. In experiments in which units were not seen to respond to i.a. drug administration, the position of the cannula at the aortic bifurcation was confirmed visually at the end of the experiment. Capsaicin (20 μM, Tocris, Bristol, UK) or cinnamaldehyde (80 mM, Sigma Aldrich, Gillingham, UK) was administered in a 100 μl bolus (10% ethanol, 10% Tween 80, 80% Saline) washed into the hindpaw with 400 μl of heparinised saline (50 Uml^−1^). Ruthenium Red (Sigma Aldrich, UK) was delivered in saline by the same route, washed in with heparinised saline.

### Agonist: temperature interactions in cutaneous afferents in naïve and inflamed rats

In the first set of teased fibre experiments the effect of RF temperature on cinnamaldehyde and capsaicin evoked activity was determined. Multifibre recordings were made, though filaments were teased sufficiently to distinguish single units using individual waveform analysis in Spike 2 v5 (CED, UK). As TRPA1 and TRPV1 are found in slowly conducting polymodal nociceptors (Story et al., 2003; Dunham et al., 2008), the units studied were most likely derived from this population.

The hindpaw was inserted into a water bath of dimensions circa 200×100×60 mm^3^ (length×width×depth) through a slit in the wall of the bath which was then made water tight with silicon dental impression material (Xantopren; Heraeus Kulzer, Hanau, Germany). The temperature of the paw, including the RF of the units under study was adjusted via alteration of the temperature of the water bath, which was measured using a thermocouple placed within the bath. Neutral-warm (30–40 °C), cool (20–25 °C) or cold (3–15 °C) water was placed into the bath. A range of temperatures was used to allow for the determination of correlative relationships between agonist responses and RF temperature. Ten minutes after the temperature change a TRP channel agonist was injected via the close arterial cannula. Agonist-evoked activity was calculated by subtracting activity in the 60 s prior to injection from the activity 60 s after injection as previously described (Dunham et al., 2008). In each experimental paradigm, the group of units under study was exposed to TRP agonist at each of cool, cold or neutral-warm temperatures, the order of which was counterbalanced across experiments. The concentrations of cinnamaldehyde and capsaicin used do not result in tachyphylaxis of response on repetitive exposure, in the majority of units (Szolcsanyi et al., 1988; Dunham et al., 2008). Desensitisation was only seen occasionally in response to capsaicin exposure, and never as a result of cinnamaldehyde exposure. If desensitisation to capsaicin was seen, the unit was excluded from the study.

Agonist: temperature interactions were studied in both naïve animals and a group of animals that had cutaneous inflammation of the hindpaw induced 3 days prior to electrophysiology via two, 50 μl CFA (complete Freund's adjuvant) injections (1 mg/ml) one each on the medial and lateral side of the ankle with CFA (Sigma Aldrich, Gillingham, UK), under halothane anaesthesia (in 2–4% O_2_) (Donaldson et al., 1993).

### TRP channel block of cold and TRPA1 agonist responses in cutaneous primary afferents

The effect of Ruthenium Red, a TRPV1/TRPA1 channel blocker (which also blocks other channels) (Story et al., 2003; St Pierre et al., 2009), was determined on both agonist (cinnamaldehyde) and cooling evoked activity in Aδ and C fibre cutaneous afferents. Filaments were finely teased and individual units identified via monopolar electrical stimulation of their RF (up to 100 V, 0.5 ms duration) enabling their conduction velocities (c.v.'s) to be calculated. C fibres had c.v.'s <1 ms^−1^ and Aδ had c.v.'s between 5 and 15 ms^−1^ as determined from compound action potential recordings, as described previously (Dunham et al., 2008). Following RF identification and calculation of c.v., a second Peltier device with smaller contact area (built in-house, surface area of circa 8 mm^2^) was used to apply cold stimuli to the RF. The Peltier was placed on the RF in firm contact with the skin. The Peltier contact only rarely evoked activity and when this occurred, the Peltier was repositioned to eliminate this before thermal stimulation. This served to ensure that cold-evoked activity was not contaminated with mechanically-evoked activity. The Peltier temperature was ramped from a holding temperature of 30 °C–5 °C over a period of 20 s.

C-cold units (Hensel, 1981) were encountered but were not included in this study as we have shown that these units do not express TRPA1 (Dunham et al., 2008). These units were identified by their characteristic ongoing bursting discharge at room temperature, in the absence of any stimulus (Hensel and Zotterman, 1951; Hensel et al., 1960).

After characterisation the cooling ramp was applied to determine the cold sensitivity of the unit. Activity evoked during this ramp was quantified simply as the total number of spikes. At the end of the cooling ramp the Peltier returned to 30 °C rapidly. A bolus of cinnamaldehyde (80 mM in 100 μl) was injected and agonist-evoked activity was quantified as described above. After a period of 5 min and after washing the arterial cannula with heparinised saline, 0.1 mM Ruthenium Red in 100 μl saline was injected as a bolus. Cinnamaldehyde was injected as before, 60 s after Ruthenium Red (Dunham et al., 2008), and the RF cooled 60 s after the cinnamaldehyde was given. The entire stimulation protocol was then repeated 5 min later for a third and final time after the injection of 1 mM Ruthenium Red.

Data are presented as mean±SEM unless stated otherwise. All statistical calculations were performed in GraphPad Prism 4.00 for Macintosh or PC (GraphPad Software, San Diego, CA, USA, available at: http://www.graphpad.com). Statistical tests are as described in the figure legends. Null hypotheses were rejected if *P*<0.05.

## Results

### TRPA1 agonist effects on thermal withdrawal thresholds

**Fig. 1:**
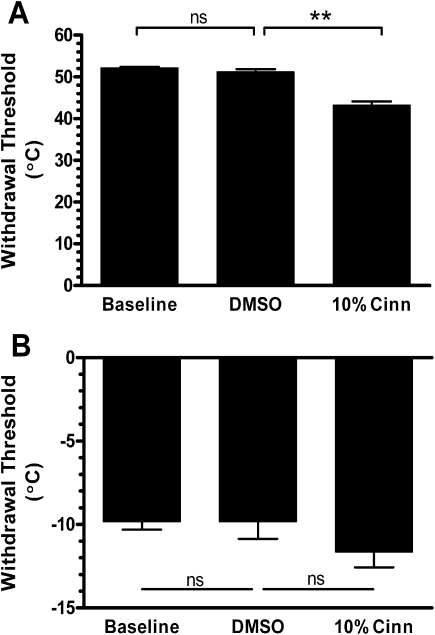
Cinnamaldehyde enhances heat sensitivity but not cold sensitivity. (A) Slow heat ramps evoked withdrawal at 52 °C and this was not changed after dimethylsulphoxide (DMSO) (100%) vehicle. Topical cinnamaldehyde (10% in DMSO) caused a significant reduction in withdrawal threshold (data from three animals with up to three ramps per treatment, ** *P*<0.01, 10% cinn vs. DMSO). (B) Noxious cold evoked withdrawal at −10 °C and this was also not affected by DMSO (100%) treatment. Cinnamaldehyde did not cause an increased sensitivity to cold (data from three animals with up to three ramps per treatment, n.s.=not significantly different).

Slow rates of contact skin heating preferentially activate C fibre, capsaicin sensitive, nociceptors and evoke withdrawal in anaesthetised rats (McMullan et al., 2004; Leith et al., 2007). A sensitisation of this reflex to heat is seen following application of capsaicin to the hindpaw skin (McMullan et al., 2004; Leith et al., 2007). The skin: lamp interface temperature that evoked EMG activity in the biceps femoris (the nociceptive withdrawal threshold), was 52.2±0.2 °C, a value equivalent to that seen in previous studies using the same method (McMullan et al., 2004; Leith et al., 2007). The withdrawal threshold was not affected by vehicle application to the skin. Cutaneous cinnamaldehyde resulted in a significant reduction in the withdrawal temperature to 43.2±0.9 °C indicating sensitisation of the reflex response to heat (Fig. 1A). This reduction in withdrawal temperature was equivalent to that seen on capsaicin application to the hindpaw skin, in previous studies (McMullan et al., 2004; Leith et al., 2007).

Cooling the hindpaw evoked reflex withdrawal at a skin: interface temperature of −9.8±0.5 °C. The cold withdrawal threshold was not affected by application of the vehicle, DMSO. Cinnamaldehyde did not enhance sensitivity to cold; on the contrary, there was a non-significant tendency for a desensitisation to cold stimuli after cinnamaldehyde application (Fig. 1B).

### Agonist: temperature interactions in cutaneous afferents in naïve and inflamed rats

**Fig. 2:**
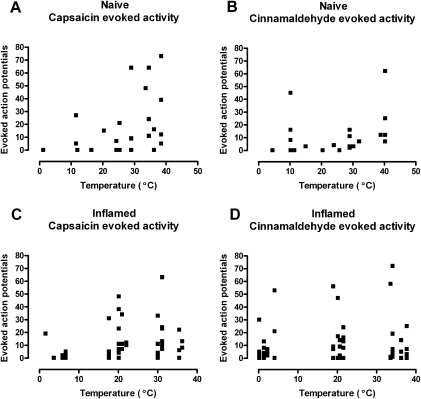
Capsaicin, but not cinnamaldehyde, evoked activity is temperature sensitive. This relationship is not altered by complete Freund's adjuvant (CFA) induced cutaneous inflammation. (A) In naïve rat skin increasing temperature increased the activity evoked by capsaicin (data from four animals, total of 10 units, activity of each recorded at three different temperatures. Spearman's correlation *r*=0.52, *P*<0.01). (B) In contrast, increasing temperature did not affect the activity evoked by cinnamaldehyde (data from four animals, 8 units each at three different temperatures. Spearman's correlation *r*=0.34, *P*>0.05). (C) Three days after CFA induction of cutaneous inflammation, increasing temperature still increases activity evoked by capsaicin (data from four animals, total of 17 units tested at three different temperatures. Spearman's correlation *r*=0.34, *P*<0.01). (D) Three days after CFA induction of cutaneous inflammation, increasing temperature still did not affect cinnamaldehyde evoked activity (data from four animals, total of 19 units each tested at three different temperatures. Spearman's correlation *r*=−0.09, *P*>0.05).

Capsaicin activates the archetypal heat sensitive TRP channel TRPV1, and TRPV1 agonists sensitise primary afferent neurones to heat stimulation (Zimmermann et al., 2005). As expected, the capsaicin-evoked activity was related to RF temperature, with evoked responses being greater at warmer RF temperatures (Fig. 2A). In contrast, the activity evoked by cinnamaldehyde was not correlated with temperature (Fig. 2B). Our hypothesis was that, if TRPA1 was modulated by cold, then a negative relationship would be apparent between RF temperature and agonist-evoked activity in TRPA1-expressing afferents. If anything the correlation between cinnamaldehyde-evoked activity and RF temperature was slightly positive. As it has been hypothesised that a temperature dependence of TRPA1 may be revealed only under pathological conditions (Reid, 2005), we also examined this correlation in afferents innervating inflamed skin. Three days after CFA cutaneous inflammation, there was again a positive, and significant correlation between capsaicin evoked activity and RF temperature (Fig. 2C). Acute inflammation did not result in a change in the relationship between cinnamaldehyde-evoked activity and temperature, which again showed no correlation (Fig. 2D).

### TRP channel block of cold and TRPA1 agonist responses in cutaneous primary afferents

We previously conducted a survey of afferents to determine sensitivity to cinnamaldehyde and the properties of these afferents have been previously published (Dunham et al., 2008). Of 72 slowly conducting afferents surveyed in normal and CFA inflamed skin, cinnamaldehyde evoked activity in 29, and of these 29 afferents, only eight also responded to cold. Thus the concordance of functional TRPA expression and cold responsiveness in rat cutaneous afferents was low, representing ∼11% of all afferents surveyed (see data in Dunham et al., 2008). In this subsequent study, only five cold sensitive cutaneous afferents were identified, only two of which responded to cinnamaldehyde. Thus cutaneous afferents that respond to cold (rather than cool) and that also respond to TRPA1 agonists are extremely rare in the rat hindpaw.

**Fig. 3:**
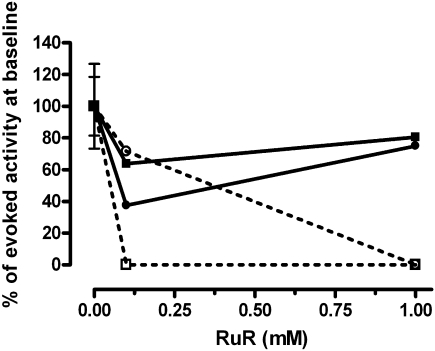
Ruthenium Red (RuR) inhibits cinnamaldehyde but not cooling evoked activity in 2A delta nociceptors. The two units are represented by either a square or circle. Cinnamaldehyde evoked activity is shown by the dotted line. Cooling evoked activity is shown by the black line. To aid comparison both cooling evoked activity and cinnamaldehyde evoked activity have been normalised to baseline (0 mM Ruthenium Red, 100%).

In these two TRPA-expressing afferents (both of which conducted in the Aδ range and had a von Frey hair mechanical threshold of 4 g), increasing concentrations of Ruthenium Red dose-dependently inhibited the cinnamaldehyde-evoked activity (as we have previously reported; (Dunham et al., 2008)). In both TRPA1-expressing Aδ units, activity evoked by RF cooling from 30 to 5 °C (using a contact Peltier device) was unaffected by Ruthenium Red (Fig. 3) even when cinnamaldehyde-evoked activity was inhibited in the same units with the same blocker. Ruthenium Red did not block cooling responses in the 3 U that did not express TRPA1 (data not shown).

## Discussion

Agonists and temperature both influence thermally sensitive TRP channels; each acts to modulate activity evoked by the other. This is a familiar concept to most—discomfort from a spicy meal can be somewhat reduced with a cool drink, i.e. the reduction in temperature is reducing the activity evoked by the capsaicin acting at TRPV1. Equally, a cool drink can feel very cold after using a menthol containing toothpaste; the temperature perception is enhanced by the action of menthol at TRPM8. Experimentally, this is evident by the modulation of channel open probability by agonist and temperature (Babes et al., 2002; McKemy et al., 2002; Peier et al., 2002).

We hypothesised that if TRPA1 is a cold sensor/transducer *in vivo*, then agonist sensitisation of TRPA1 would also sensitise TRPA1 to cold stimulation, in just such a manner as exists *in vitro* for TRPM8 (McKemy et al., 2002; Peier et al., 2002). It is however, very difficult to generate an isolated noxious cold stimulus in order to examine this phenomenon *in vivo*. Between the baseline temperature and the target noxious temperature are, unavoidably, innocuous cool temperatures. Cool and cold stimuli evoke different behaviours in laboratory rodents, and noxious cold threshold is difficult to determine, as animals will briefly withdraw from a cool stimulus (J.P. Dunham, unpublished results). In addition there is some debate about where the transition between uncomfortable cold and painful cold lies, i.e. how to define noxious cold threshold. The cooling receptor TRPM8 has a wide activation temperature range in neurones (31–27 °C) (McKemy et al., 2002; Peier et al., 2002), which correlates closely with human psychophysical cooling thresholds (Harrison and Davis, 1999; Meier et al., 2001; Davis and Pope, 2002). Although 15 °C is often cited as the noxious cold threshold in humans (Davis and Pope, 2002), noxious cold thresholds are often more variable than noxious heat thresholds in psychophysical experiments in man (Foulkes and Wood, 2007) and are often very difficult to ascertain accurately (Davis, 1998). Cold pain thresholds have been reported as being between 6 and 23 °C depending on measurement method, and site and rate of cooling (Chen et al., 1996; Davis, 1998; Harrison and Davis, 1999; Meier et al., 2001). In two reports, cold pain was not consistently reported by subjects until stimulus temperature was below 10 °C (Chen et al., 1996; Davis, 1998), and in a third study pain threshold was reported as 0 °C (Strigo et al., 2000) in ambient environments (25 °C). Cold pain thresholds are dependent on the rate of skin cooling, being lower when cooling rates are faster (Harrison and Davis, 1999; Strigo et al., 2000). Indeed the report that “pain was clearly reported when temperatures fell below ∼10 °C” (Davis, 1998) and earlier studies (see references in; (Davis, 1998)) suggest that cold pain threshold is lower than that commonly cited.

To overcome this problem, we used electromyography in anaesthetised animals to measure withdrawal threshold to a noxious cold stimulus. Heat evoked flexor withdrawal (captured via electromyogenic activity in a limb flexor) in anaesthetised rats is a well established response evoked by activation of heat sensitive nociceptors (Yeomans et al., 1996; Yeomans and Proudfit, 1996). In this way, the measured response (EMG) could be largely attributed to nociceptor and not to non-nociceptor activity. Noxious cold withdrawal occurred only at very low skin surface temperatures in the anaesthetised rat, and a TRPA1 agonist did not sensitise the withdrawal reflex to cold. If TRPA1 is a cold transducer, we hypothesised that a TRPA1 agonist would enhance a response evoked by noxious cold, which was not the case. These data are supported by the findings that (1) cinnamaldehyde does not affect cold pain thresholds in humans (Namer et al., 2005, 2008), and (2) mustard oil does not sensitise spinal dorsal horn neurones to peripheral cold stimulation (Sawyer et al., 2009), and (3) ablation of TRPV1/TRPA afferents with resiniferatoxin in adult rats enhances cold sensitivity (Pecze et al., 2009).

It is surprising that the skin surface temperatures required to evoke a withdrawal were so cold, ∼−10 °C, given previous studies of cold withdrawal threshold in awake animals. It is possible that extremely cold surface temperatures might result in tissue damage due to freezing, which is known to be very painful (Beise et al., 1998). Cold stimuli were given at 10 min intervals, but if tissue damage due to freezing occurred at each stimulus, one would expect to observe a sensitising effect of the multiple stimuli over time. This was not seen at all, in fact withdrawal temperatures were remarkably consistent throughout the trials. We do not, therefore believe that this stimulus resulted in tissue damage as sensitisation was not seen.

In contrast, TRPA1 agonist did result in sensitisation to noxious heat. The slow heat ramps used in these studies preferentially activate C fibre nociceptors expressing TRPV1 (McMullan et al., 2004), many of which also express TRPA1 (Story et al., 2003; Dunham et al., 2008). There are known interactions between TRPV1 and TRPA1 (Bandell et al., 2004; Bautista et al., 2006; Jeske et al., 2006; Akopian et al., 2007; Ruparel et al., 2008), and cinnamaldehyde-evoked heat sensitisation is initiated by TRPA1 but affected by TRPV1 (Bandell et al., 2004). Our data, generated using the slow heat ramp to preferentially activate C fibre heat sensitive nociceptors, confirm that TRPA1-mediated sensitisation to heat is indeed mediated by the C fibre heat sensitive nociceptors, as one would expect from the expression profile of TRPA1 (Kobayashi et al., 2005). These findings also confirm that TRPA1 agonist is reaching cutaneous afferents in these studies, showing that the lack of cold sensitisation cannot be attributed to poor access of the cinnamaldehyde to the afferent terminals.

The data using thermally-evoked noxious withdrawal do not therefore support our first hypothesis, that a TRPA1 agonist would enhance responses evoked by noxious cold. We tested this hypothesis further by measuring agonist evoked activity in single identified nociceptors at different RF temperatures. For TRPV1, agonist-evoked activity was indeed enhanced at higher temperatures *in vivo*, as predicted. In both normal and inflamed skin, warming the RF of capsaicin sensitive afferents enhanced the activity evoked by capsaicin, just as one would expect from the experience of eating a hot curry. In contrast the activity evoked by cinnamaldehyde was not influenced by RF temperature. Reid (2005) postulated that pathology may reveal a temperature sensitivity in TRPA1 such that the channel is temperature insensitive (or less temperature sensitive) under normal conditions yet in pathology the channel becomes cold responsive. This hypothesis gained support from studies in which antisense oligodeoxynucleotides or TPRA1 antagonists had no effect on cold behaviours in normal animals but reduced the cold hypersensitivity seen after nerve injury (Katsura et al., 2006) or inflammation (Petrus et al., 2007). We could not demonstrate any temperature modulation of cinnamaldehyde-evoked activity in single afferent fibres level, even 3 days after CFA induced inflammation, at which time TRPA1 is known to have been upregulated (Dunham et al., 2008).

An alternative possibility is that TRPA1 might transduce cold stimuli but that this function is somehow distinct from its agonist sensitivity. This would not be consistent with the interaction of thermal and agonist stimuli at of closely related channels such as TRPV1 and TRPM8, but the possibility must be considered when trying to untangle the various data regarding TRPA1's postulated role in cold transduction. The study of both cold and TRPA1-agonist-induced responses in single afferents proved problematic as afferents in which responses were evoked by both stimuli were not common. In the two afferents identified in this study, both, interestingly, Aδ, and not C fibre nociceptors, both cold and TRPA1 agonist responses could be evoked. Ruthenium Red is a non-competitive TRP channel blocker that blocks both TRPA1 and TRPV1 (Story et al., 2003), and in TRPV1, binds within the channel pore to produce channel block (Garcia-Martinez et al., 2000). Thus, if TRPA1 carries a cold evoked current *in vivo*, Ruthenium Red would be predicted to inhibit it, as it inhibits agonist-evoked activity *in vivo* (Ji et al., 2007; Dunham et al., 2008). Although agonist responses could be blocked, the cold evoked activity in the same fibres was not affected, even at very high (1 mM) concentrations of Ruthenium Red.

It was recently observed that although TRPA1 plays a role in cold sensing in visceral sensory neurones, it has little role in somatic noxious cold detection (Fajardo et al., 2008). Our data using different experimental approaches *in vivo* support these *in vitro* findings, and do not support that hypothesis that TRPA1 is a cold transducer in rat somatic primary afferents *in vivo*.
